# Nanoscale dynamics of enhancer–promoter interactions during exit from pluripotency

**DOI:** 10.1093/nar/gkaf1255

**Published:** 2025-12-08

**Authors:** Gabriela Stumberger, David Hörl, Dimitra Tsouraki, Clemens Steinek, A Marieke Oudelaar, Heinrich Leonhardt, Hartmann Harz

**Affiliations:** Human Biology & Bioimaging, Faculty of Biology, Ludwig-Maximilians-Universität München, Munich 81377, Germany; Human Biology & Bioimaging, Faculty of Biology, Ludwig-Maximilians-Universität München, Munich 81377, Germany; Genome Organization and Regulation, Max Planck Institute for Multidisciplinary Sciences, Göttingen 37077, Germany; Human Biology & Bioimaging, Faculty of Biology, Ludwig-Maximilians-Universität München, Munich 81377, Germany; Genome Organization and Regulation, Max Planck Institute for Multidisciplinary Sciences, Göttingen 37077, Germany; Human Biology & Bioimaging, Faculty of Biology, Ludwig-Maximilians-Universität München, Munich 81377, Germany; Human Biology & Bioimaging, Faculty of Biology, Ludwig-Maximilians-Universität München, Munich 81377, Germany

## Abstract

While compelling genetic evidence supports the role of enhancers in regulating promoter activity even over large genomic distances, it remains unclear to what extent physical proximity to promoters is required. To address this, we combined fluorescence *in situ* hybridization (FISH) with super-resolution microscopy and Tri-C to examine enhancer–promoter (E–P) distances and regulatory element clustering at regulated loci (*Nanog, Dppa3, Dnmt3a, Sox2, Prdm14*) during the transition from naive to primed pluripotency in mouse embryonic stem cells. Despite transcriptional changes of several orders of magnitude, most genes show no major alterations in median E–P distances or in the probability of multiway contacts across states. However, Tri-C reveals a weak enrichment of multiway contacts at *Nanog* in naive cells, where it is highly expressed. Because transcription often occurs in transient bursts within a subset of cells, we combined RNA and DNA FISH to identify active alleles. For *Nanog* and *Dppa3*, reduced E–P distances correlate with transcriptional activity. Together, these findings support models in which transcription is associated with transient E–P proximity and suggest that multiway contact formation among regulatory elements may contribute to gene regulation.

## Graphical Abstract

**Graphical Abstract:**
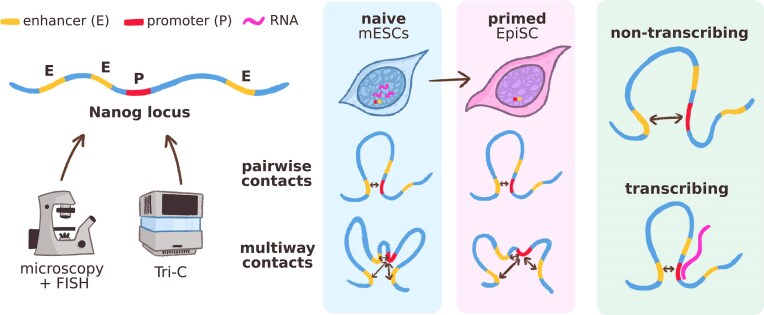

## Introduction

In recent years, it has become clear that in addition to genetic elements such as enhancers and transcription factors, physical aspects such as the spatial and temporal proximity of these elements also regulate transcription, which is important for disease [1] and development [2]. While it is commonly accepted that enhancer–promoter (E–P) interactions can drive gene expression through the recruitment of transcription factors and RNA Pol II, several conflicting models describe how regulatory elements communicate (reviewed in [3]).

On the one hand, action-at-a-distance models propose that direct physical contact between enhancers and promoters is not required for a functional interaction. This view is supported by imaging studies, which report average E–P distances of ∼200–350 nm [4–11] at transcriptional activation, and by observations showing no [4, 6, 12, 13] or even inverse correlation between E–P proximity and transcription at the respective locus [11, 14]. Some variants of these models suggest that E–P communication occurs via phase-separated compartments enriched in transcription factors, coactivators, and RNA polymerase II, ranging several hundred nanometers in diameter [6, 15–19]. Alternatively, the transcription factor activity gradient model posits that enhancer-bound coactivators (e.g. p300) modify nearby transcription factors (e.g. acetylation), creating a gradient of active TFs diffusing from the enhancer [20]. On the other hand, structural studies of various components involved in transcription (eg. Mediator–Pol II pre-initiation complex) [21–24] and measurements showing reduced E–P distances upon transcriptional activation [9, 10, 25–28], support a direct-contact model, in which enhancers physically interact with promoters over short spatial distances. This discussion is further complicated by the fact that eukaryotic genes, especially those encoding developmental regulators, are typically regulated by multiple enhancers. Recent studies have suggested the existence of multi-enhancer hubs or nested microcompartments, where several (genomically distant) *cis* regulatory elements cluster in close spatial proximity to each other, activating transcription [29−31]. However, it is still unknown how frequent these multiway hubs are in single cells and whether they play any role in regulating gene expression. Another subject of discussion is the duration of communication between the respective elements. While classical studies assume stable loops, live cell studies show that the interactions of chromatin elements are dynamic, with contact durations in the range of 10–30 min, possibly even significantly shorter in the case of E–P interactions (reviewed in [3]).

Both sequencing-based and microscopic methods for studying genome architecture have undergone significant advances in recent years [27, 32–42] and have led to a deeper understanding of genome organization. These two methodological approaches are complementary: while microscopy can provide single-cell data, sequence-based methods enable the detection of rare events within large populations.

Here, we utilize oligo-based DNA fluorescence *in situ* hybridization (FISH) combined with super-resolution microscopy and Tri-C, to investigate E–P 3D distances of five differentially expressed genes (*Nanog, Dppa3, Dnmt3a, Sox2, Prdm14*) during the naive to primed transition in mouse embryonic stem cells (mESCs). This developmental transition is known to involve extensive epigenetic reprogramming and conformational changes at regulatory elements [43–46]. We observe that for most, but not all measured genes, both pairwise and multiway E–P conformations undergo only minor changes in distance and contact frequency between naive and primed states. Tri-C data reveal a modest enrichment of multiway contacts at the *Nanog* locus in naive cells, where this protein is highly expressed. Furthermore, we could show that at the *Nanog* and *Dppa3* loci, actively transcribed alleles display significantly shorter E–P distances, supporting the notion that spatial interactions between enhancers and promoters are transient.

## Materials and methods

### Cell culture

Naive J1 mESCs were cultured in serum-free media consisting of: N2B27 [50% neurobasal medium (Life Technologies), 50% Dulbecco’s modified Eagle’s medium (DMEM)/F12 (Life Technologies)], 2i [1 μM PD032591 and 3 μM CHIR99021 (Axon Medchem)], 1000 U/ml recombinant leukemia inhibitory factor (LIF; Millipore), and 0.3% bovine serum albumin (BSA; Gibco), 2 mM L-glutamine (Life Technologies), 0.1 mM B-mercaptoethanol (Life Technologies), N2 supplement (Life Technologies), and B27 serum-free supplement (Life Technologies). Naive mESCs were cultured on 0.2% gelatin-coated flasks.

To derive primed mSCs, naive mESC were plated on Geltrex (Gibco) diluted 1:100 in DMEM/F12 medium (Gibco) and transferred to the same serum-free media used for naive mESCs, without 2i, LIF, and BSA and supplemented with 10 ng/ml Fgf2 (R&D Systems), 20 ng/ml Activin A (R&D Systems), and 0.1× Knockout Serum Replacement (Life Technologies). Cells were differentiated for 7 days, splitting every 2–3 days. All cells were tested negative for Mycoplasma contamination by polymerase chain reaction (PCR).

### Quantitative real-time PCR

To validate the identities of naive and primed cell types and confirm the transcription levels of selected pluripotency genes, quantitative real-time PCR (qRT-PCR) was performed (Supplementary Fig. S1C). Total RNA was isolated using NucleoSpin RNA, Mini kit for RNA purification (Marcherey-Nagel) according to the manufacturer’s instructions. cDNA was synthesized using the High-Capacity complementary DNA (cDNA) Reverse Transcription Kit (Applied Biosystems) with 500 ng RNA as input. qRT-PCR with primers (Supplementary Table S1) was performed in 10 μl reactions with 5 ng cDNA as input. Luna^®^ Universal qPCR Master Mix (New England Biolabs) was used for detection. The reactions were run on a LightCycler480 (Roche).

### Immunofluorescence for validation of cell identity

To further validate the identities of naive and primed cell types at a single-cell level, immunofluorescence of four key marker genes was performed (Supplementary Fig. S1A and B). NANOG and ESRRB were used as naive markers, together with OTX2 and OCT6 as primed markers [47–52]. Cells were seeded on Geltrex (Gibco) coated coverslips (12 × 12 mm) at a density of 10^5^ cells per cm^2^ on the previous evening. The following steps were performed at room temperature. Next morning, cells were washed 2× with 1× Dulbecco’s Phosphate buffered saline (PBS) and fixed with methanol-free 4% formaldehyde (Polysciences, 18814-20) in 1× PBS for 10 min. Cells were rinsed with 1× PBS and washed with 1× PBS for 5 min. Cells were permeabilized with 0.5% Triton X-100 (Sigma–Aldrich) in 1× PBS for 15 min, rinsed with 1× PBS and then washed with 1× PBS. Cells were blocked in 2.5% BSA for 1 h and incubated with primary antibodies for 1 h. The samples were washed 3× for 5 min with 1× PBST and incubated for another 1 h with secondary antibodies. Samples were washed 3× for 5 min with 1× PBS with 0.2% Tween 20 (Carl Roth) (PBST). DNA was counterstained with DAPI (4′,6-diamidino-2-phenylindole) (1 μg/ml in 1× PBS) for 5 min and washed 2× with 1× PBS. Slides were mounted in Mowiol (2.5% DABCO (1,4-diazabicyclo [2.2.2]octane), pH 8.5), dried for 30 min and sealed with nail polish. For a list of used antibodies, see Supplementary Table S2.

### Processing published raw sequencing data for putative enhancer calling model input

To obtain the .bam files required for the activity-by-contact (ABC) -model input, the data needed to be reprocessed. For ATAC-seq (GEO: GSE131556) and H3K27ac data (GEO: GSE156261) [48] the raw sequences were downloaded from GEO [53, 54] using SRA-Toolkit [55] For ATAC-seq, sequences were quality trimmed using TrimGalore (zenodo.org/records/7598955), discarding reads shorter than 15 bp. Reads were aligned to mm9 using bowtie2 [56] with parameters ‘–very-sensitive –trim3 1 -X 2000’. Mitochondrial reads and PCR duplicates were removed using Piccard tools (broadinstitute.github.io/picard/). Peaks were called using Genrich (github.com/jsh58/Genrich) in ATAC-seq mode, with otherwise default parameters. Bigwig files for visualization were created using deepTools [57] bamCoverage with ‘–binSize 10 –normalizeUsing RPKM’.

Raw H3K27ac ChIP-seq sequences were quality controlled with FastQC v0.12.1(www.bioinformatics.babraham.ac.uk/projects/fastqc/) and the output was summarized with MultiQC [58] Reads were aligned to the mm9 genome using bwa mem 0.7.10-r789 [59] using default parameters. Mitochondrial reads, Y chromosome, and nonchromosome scaffolds were removed. Bigwig files for visualization were created using deepTools bamCoverage with ‘–binSize 30 –normalizeUsing RPKM’.

### Putative E–P pair calling

Putative E–P pairs in naive and primed cells were called using the ABC model [60] with default parameters. The epigenetic signal was normalized to the K562 data provided in the model. As input, already published ATAC-seq (GEO: GSE131556), H3K27ac ChIP-seq (GEO: GSE156261), RNA-seq (GEO: GSE131556) [48], and Hi-C (GEO: GSE124342) [61] data from naive and primed mESCs was used. Genes and transcription start sites were annotated as described in [60]. mm9 blacklisted regions from [62] were used. The generated datasets and interactions were visualized using CoolBox [63]. A complete list of all called enhancers in the genome will be provided on request.

### Target selection

Genes were first filtered for developmental genes which show significant changes in gene expression during the differentiation from naive to primed mouse stem cells. Genes with at least three putative enhancers over both states were then selected. It is important to note that the selected enhancers are putative and do not represent a comprehensive list of all enhancers of a gene. For chosen E–P pairs see Supplementary Tables S2 and S3 and Supplementary Fig. S2. For target coordinates (mm9) see Supplementary Table S4. For information on which of the chosen enhancers have previously been functionally validated see Supplementary Table S2.

### Probe design

For oligoDNA FISH probes, a 20 kb region around each target was tiled into nonoverlapping 40 bp oligonucleotides. These were filtered for uniqueness against the mm9 genome using BLAT [64] (default settings), retaining those with <5 matches and excluding repetitive sequences. Melting temperatures were constrained to 30–60°C in 50% formamide. This approach was tailored to a higher probe density [65], compared to other published design tools, optimized for whole-genome coverage or chromosome walking [66−68]. A 20 bp barcode was appended to the 3′ ends for visualization via fluorescently labeled readout oligos. RNA SABER FISH probes against introns were designed with PaintSHOP [69]. A complete list of probe sequences can be found in Supplementary Table S5 and S6.

### Probe synthesis

DNA FISH probes were synthesized as described previously [67, 70] with adaptations. Briefly, the target oligos were amplified from the template oligopool (GenScript) via PCR using 20 nt primers (obtained from PaintSHOP, ordered from Merck), according to the manufacturer’s instructions (BIO-21110, Bioline). The PCR product was purified using columns (#740609, Macherey-Nagel).

The purified DNA was converted to RNA via a high yield *in vitro* transcription (IVT) according to the manufacturer’s instructions (#E2050S, New England Biolabs). Each 30 μl reaction consisted of ∼1 μg template DNA, 6.66 μM of each NTP, 20 U/μl RNaseOUT (Thermo Fisher) and 2 μl T7 polymerase mix. To maximize yield, the reaction was incubated at 37°C for 16 h.

DNA was removed by incubating the product from the IVT reaction with 2 μl DNase I (2 U/μl, M0303S, New England Biolabs) and 20 μl RNAse-free water at 37°C for 15 min. The RNA was purified using columns (#T2010, New England Biolabs) and converted to DNA via a reverse transcription (RT) reaction. Each 30 μl RT reaction contained 1/5 of the purified RNA from IVT, 2 μM of 41 nt froward RT primer with readout barcode, 1.5 mM each dNTP, 300 U Maxima H Minus Reverse Transcriptase (#EP0751, Thermo Fisher), and 1× RT buffer. The reaction was incubated at 50°C for 1 h and inactivated at 85°C for 15 min.

The template RNA was removed by incubating the product from the RT reaction with 20 μl of each 0.5 M ethylenediaminetetraacetic acid (EDTA) and 1 M NaOH at 95°C for 15 min. The DNA product was purified using columns (#T1030L, New England Biolabs) and eluted in 15 μl ultra-pure H_2_O and stored at −20°C. The quality of the probes was assessed using NanoDrop and poly-acrylamide gel electrophoresis.

SABER RNA FISH probes were synthesized as described in [71]. NOVA FISH probes were synthesized as described in [41].

Fluorescently labeled readout oligonucleotides were ordered from Eurofins/Ella Biotech. DNA FISH readout oligos were labeled with either Atto565 (spinning disk confocal microscopy) or Atto594 [stimulated emission depletion (STED) microscopy] for promoters and STAR635P for enhancers. Readout oligos for RNA FISH were labeled with Atto594.

### Fluorescence *in situ* hybridization

FISH was performed as described previously [65, 72], with minor modifications. Cells were seeded on Geltrex (Gibco) coated coverslips (12 × 12 mm) at a density of 10^5^ cells per cm^2^ on the previous evening. Next morning, cells were washed 2× with 1× Dulbecco’s PBS and fixed with methanol-free 4% formaldehyde (Polysciences, 18814-20) in 1× PBS for 10 min. Cells were rinsed with 1× PBS and washed with 1× PBS for 5 min. Cells were permeabilized with 0.5% Triton X-100 (Sigma–Aldrich) in 1× PBS for 15 min, rinsed with 1× PBS and then washed with 1× PBS.

For oligoDNA FISH only, cells were treated with 0.1 M HCl for 5 min and washed 2× with 2× saline-sodium citrate (SSC) buffer for 5 min. RNA was digested by incubating cells with 100 µg/ml RNase A (ThermoScientific) in 2× SSC for 30 min at 37°C. After washing 2× with 2× SSC, cells were pre-equilibrated in 50% formamide (Merck) in 2× SSC for 60 min. The coverslip was placed cell-side down onto 4.5 µl of hybridization solution [0.05–0.2 nM probe, 50% formamide, 10% dextran sulfate (Sigma–Aldrich), 0.125% Tween 20 (Carl Roth), 2× SSC] and sealed with rubber cement (Marabu). Slides were placed on a heat block at 81°C for 3 min and incubated at 37°C overnight (16–20 h). After incubation, coverslips were washed 2× with 2× SSC for 15 min, followed by two washes with 0.2× SSC at 56°C, 2 washes with 4× SSC and one wash with 2× SSC. The second hybridization was performed in 20 µl of secondary hybridization solution (250 nM fluorescently labeled barcode, 10% dextran sulfate, 35% formamide, 2× SSC) for 30–120 min at room temperature. Cells were then washed 1× with 30% formamide in 2× SSC for 7 min at 37°C, 2× with 2× SSC for 5 min, 1× with 0.2× SSC at 56°C, 1× with 4× SSC for 5 min, and 1× with 2× SSC for 5 min. Sample were post-fixed with 4% PFA in 2× SSC for 10 min and washed 2× with 2× SSC for 5 min. DNA was counterstained with DAPI (1 μg/ml in 2× SSC) for 5 min and washed 2× with 2× SSC. Slides were mounted in MOWIOL (2.5% DABCO, pH 8.5), dried for 30 min and sealed with nail polish. The same procedure was applied for NOVA FISH, omitting the secondary probe hybridization.

For sequential SABER RNA and oligoDNA/NOVA FISH, cells were seeded into Geltrex-coated channel slides (#80161, Ibidi) and pre-treated as described above, omitting RNA digestion and using RNase-free reagents. After permeabilization, cells were washed with 2× SSC. The slide was filled with 200 µl hybridization solution (same as for DNA FISH with 1–2 nM probe) and incubated at 42°C for 2 days. Cells were then washed 2× with 2× SSC at 37°C for 10 min, 2× with 0.2× SSC at 60°C for 5 min, 1× with 2× SSC and transferred into 1× PBS. Cells were incubated with 100 nM readout probe in 1× PBS and 1 μg/ml DAPI for 1 h at 37°C. The cells were washed with 1× PBS at 37°C for 5 min and rinsed 2× with 1× PBS. Cells were post-fixed with 4% formaldehyde and washed with 1× PBS for 5 min. To enable later alignment of RNA and DNA images, the sample was incubated with FluoSpheres (505/515 nm, #P7220, Thermo Fisher) diluted 1:50 in 1× PBS for 15 min. Nonattached beads were washed away by rinsing 3× with 1× PBS. The sample was imaged in 1× PBS with 2.5% DABCO. After imaging the RNA, the slide was rinsed with 1× PBS and treated as described in the DNA FISH protocol above. Imaging was again performed in 1× PBS with 2.5% DABCO.

### Microscopy

**Figure 1.**
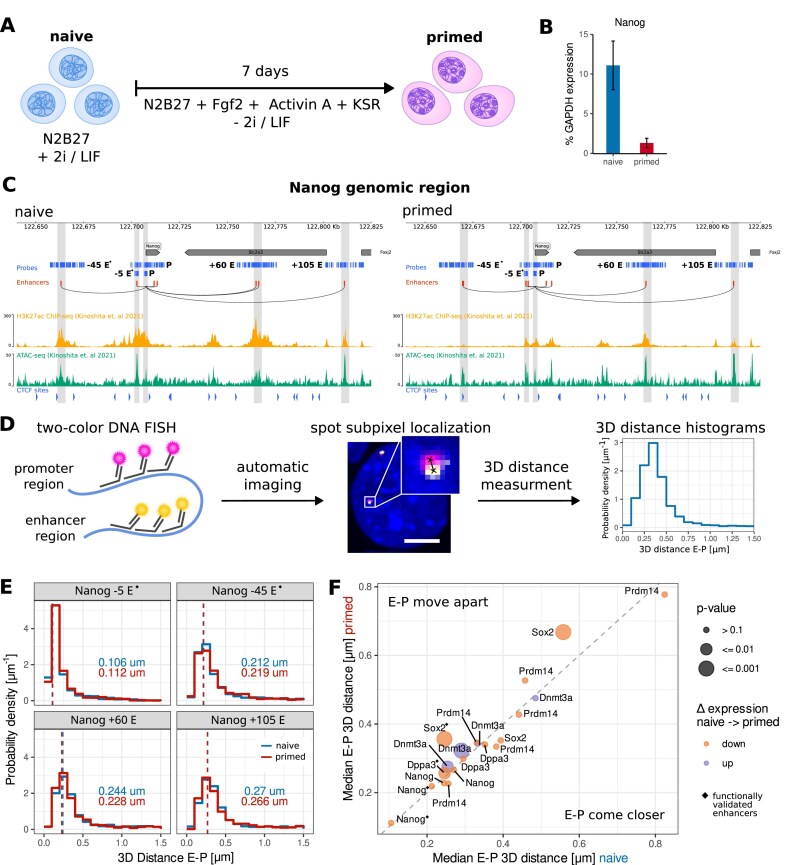
E–P distances of selected differentially expressed genes show small changes during differentiation from naive to primed mESCs. **(A)** Cell culture model of naive to primed transition in mESCs. **(B)** Changes in *Nanog* transcription levels (% GAPDH transcription) in naive and primed cells measured by qRT-PCR. The graph depicts median ± standard error of the mean (*n* = 5 biological replicates). **(C)**  *Nanog* genomic region for naive and primed cells, showing from top to bottom: DNA oligoFISH and NOVA FISH probes against promoter and selected enhancers, all predicted enhancers, connection of *Nanog* promoter to its enhancers, H3K27ac ChiP signal (from [48]), ATAC-seq signal (from [48]), CTCF binding motifs, and targeted enhancer regions (vertical gray stripes). Functionally validated enhancers are marked by ^◆^. **(D)** Experimental workflow: regions of interest are marked with two-color DNA oligoFISH, the samples are imaged automatically with spinning disk confocal microscopy, FISH spots are detected automatically with subpixel localization accuracy and 3D distances between matched E–P spots are calculated to produce a E–P distance distribution of the population. Scale bar represents 1 μm. **(E)** 3D distance [μm] distributions between *Nanog* promoter and its −5, −45, +60, and +105 (putative) enhancers in naive and primed cells. Dashed line and number next to the histogram represent the median distance. The changes between naive and primed cells are not significant (*P* > 0.05, two-sided Wilcoxon rank sum test, BH correction). From closest to furthest enhancer: *n*_naive_ = 916, 1718, 837, 1220; *n*_primed_ = 1037, 1038, 1362, 701; three biological replicates each. **(F)** Change in median 3D E–P distance [μm] of genes down- and up-regulated during the naive to primed transition. E–P pairs above the diagonal show increased distances during differentiation, while distance in pairs below the diagonal decreases. Each circle refers to a different enhancer. The test of statistical significance is the same as in panel (E). Functionally validated enhancers are marked by ^◆^.

Images of pairwise contacts relating to Fig. 1 were acquired using spinning disk confocal microscopy on a Nikon TiE, equipped with a Yokogawa CSU-W1 spinning-disk confocal unit (50 μm pinhole size), an Andor Borealis illumination unit, Andor ALC600 laser beam combiner (405/488/561/640 nm), Andor IXON 888 Ultra EMCCD camera, and a Nikon 100×/1.45 numerical aperture (NA) oil immersion objective, as also described in [65, 73]. The microscope was controlled via NIS Elements (Nikon, ver. 5.02.00). To ensure an unbiased selection of cells, most images were taken automatically, using NIS-Elements JOBS (Nikon). Images were acquired at 405, 561, and 640 nm using 10%, 75%, and 75% laser powers, with corresponding exposure times of 50, 100, and 50 ms.

**Figure 2.**
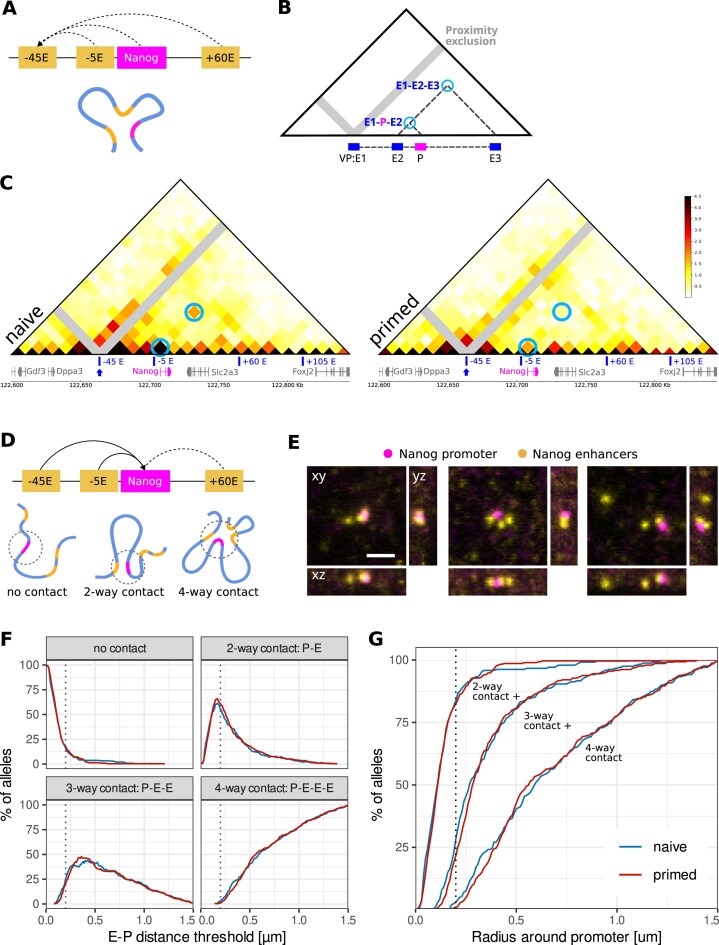
Changes in multiway E–P contacts at the *Nanog* locus during the naive to primed differentiation. **(A)** Schematic representation of Tri-C viewpoint (VP) and possible interactions at the *Nanog* locus. **(B)** Schematic representation of Tri-C data visualization. VP-specific contact matrices show the interaction frequencies at which the viewpoint interacts with two genomic regions simultaneously. Proximity signals around the viewpoint are excluded (gray stripes). **(C)** Tri-C contact matrices of the *Nanog* locus (chr6: 122,600,000–122,845,000; 10 kb resolution; mm10) in naive and primed cells. The data is shown from the viewpoint of the –45 enhancer. Contacts between 45E–P–5E, as well as between 45E–P–60E are highlighted with blue circles. **(D)** Schematic representations of possible E–P constellations: promoter does not contact enhancers; promoter contacts one enhancer at a time; promoter contacts several enhancers at the same time. **(E)** STED microscopy images (maximum intensity projections) of enhancer and promoter constellations for *Nanog* and enhancers shown in panel (D). Scale bar represents 1 μm. **(F)** Interaction frequencies between a promoter (P) and different numbers of enhancers for a range of contact thresholds [µm]. Naive cells are plotted in blue, primed in red. *n*_naive _= 240, *n*_primed _= 291 over three biological replicates. **(G)** Cumulative plot of data shown in panel (F). The curves represent interaction frequencies between a promoter and at least 1, 2, or 3 enhancers (two-way+, three-way+, or four-way contact) over a range of contact thresholds [µm].

**Figure 3.**
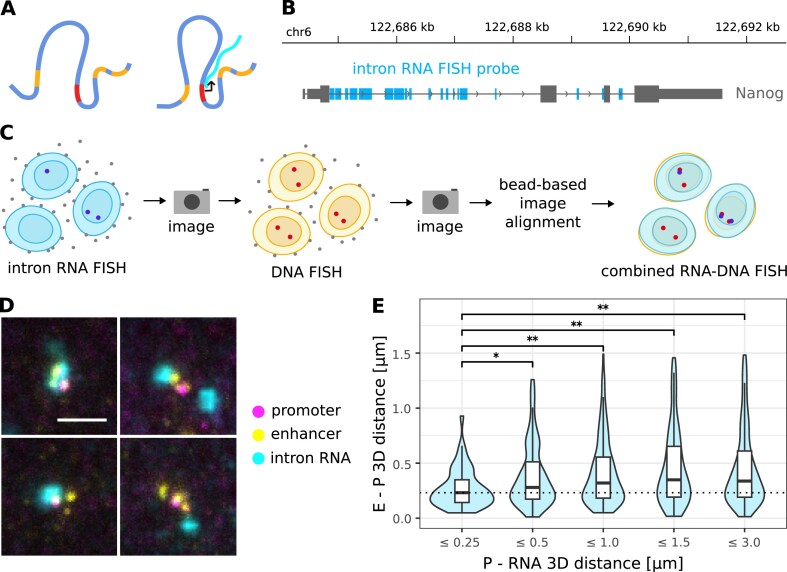
Shorter E–P distances correlate with active transcription. **(A)** Schematic representation: do enhancer and promoter come closer when the gene is actively transcribed? **(B)** Genomic location of *Nanog* intron RNA FISH probes. **(C)** Experimental workflow: intron RNA is marked by RNA FISH, imaged, promoters and enhancers are marked by DNA FISH, imaged, then RNA and DNA images are aligned using fiducial markers. **(D)** STED microscopy images (maximum intensity projections) of promoter (magenta), enhancers (yellow), and intron RNA (cyan) for *Nanog*. Scale bar represents 1 μm. **(E)** E–P 3D distance [µm] changes significantly based on distance of nascent RNA to P (*P* < 0.05: *, *P* < 0.005: **, two-sided Wilcoxon rank sum test). *n*_≤0.25_ = 46, *n*_≤0.5_ = 156, *n*_≤1_ = 272, *n*_≤1.5_ = 172, *n*_≤3_ = 259 over three biological replicates.

Imaging of experiments related to Fig. 2 was performed using STED super-resolution microscopy on a 3D STED microscope (Abberior Instruments), equipped with three pulsed excitation lasers (485, 594, and 640 nm), one pulsed depletion laser (775 nm), and Avalanche photodiodes for detection. All acquisitions were performed using a 100× UPlanSApo 1.4 NA oil immersion objective (Olympus). Imaging of Fig. 3 was performed using the same microscope in confocal mode.

To enable measurement of many E–P configurations in individual cells, we automated STED imaging using Python (version 3.7 or later) via the SpecPy (v.1.2.3) (github.com/certified-spec/specPy) interface to the Imspector microscope control software (v.16.3) [65, 74]. In short, we had our system acquire confocal overview stacks (50 × 50 × 5 µm FOV in xyz, 150 × 150 × 250 nm pixel sizes) in a regular grid or spiral. After each overview, we detected co-localizing promoter and enhancer signals and proceeded to acquire small STED stacks (2.5 × 2.5 × 2.5 µm, 30 × 30 × 150 nm pixel size) around each detection before moving on to the next overview. For samples with sequential RNA and DNA FISH, green FluoSpheres (505/515 nm, #P7220, Thermo Fisher) were imaged in all overview stacks.

### Chromatic aberration correction

Correction of chromatic aberrations for spinning disk images was done based on calibration samples of cells covered with Tetraspeck four-color fluorescent microspheres (100 nm diameter). To estimate the chromatic shift between two channels, we detected beads in both channels as local maxima above a user-defined threshold after applying a DoG filter. We then estimated subpixel coordinates of the detections by fitting a 3D Gaussian function in the local environment of the bead. As shifts were generally small, we matched bead coordinates in one channel to the closest coordinate in the other (using linear assignment to prevent matching one bead with multiple partners and discarding matches with a distance above 1 μm). The matched coordinate pairs from multiple images were pooled and affine transformation parameters between them were estimated using least-squares fitting with RANSAC outlier removal. The resulting parameter matrices (for coordinates in physical units) were saved to a JSON text file.

To correct tables of FISH spot locations in new images, we loaded the transformation matrix of each channel to a reference channel and applied it to the coordinates of that channel (detections in the reference channel were left as-is). Notably, as our microscope allows for both top-to-bottom and bottom-to-top recording of z-stacks we also saved the direction to the parameter file. If a new image did not match the z-direction of calibration images, z-coordinates were flipped around the z-center of the image before correction and flipped back afterwards.

As we did not observe strong chromatic aberrations in STED images, and they are drastically reduced due to use of a single depletion laser for both excitation channels [75], we did not apply any coordinate corrections (except to align sequential DNA- and RNA-FISH) to STED data.

### Characterization of chromatic aberrations

Using the RANSAC-inlier coordinate pairs of the 561 and 640 nm channels of the calibration images, we constructed a linearly interpolated map of the shifts (Supplementary Fig. S3A). We observe a lateral (xy) zoom aberration across the field-of-view with shifts increasing radially from approximately the center, while axial (z) shifts remain relatively constant but nonzero across the field.

Our calibration dataset consists of 11 images, allowing us to quantify the chromatic aberration correction performance via cross-validation. We proceed in a leave-one-out fashion, estimating chromatic shift from 10 of the images and using it to correct the 11th, repeating until we have corrected all coordinates. The broad distributions of lateral shifts are drastically reduced and centered around zero after correction (Supplementary Fig. S3B). The z shifts still have a broad distribution (likely due to lower localization accuracy), but the systematic shift is corrected well.

### Image analysis

FISH spots were detected in each channel with sub-pixel accuracy using RS-FISH and Gaussian fitting [76]. Parameters ‘Thershold’ and ‘intensityThreshold’ were adjusted as necessary.

For spinning disk confocal images, the spot coordinates were corrected for chromatic aberrations (see above). Nuclear segmentation masks were generated from images of DAPI-stained nuclei, using a Cellpose [77] model, trained on naive- and primed- cell nuclei. FISH spots outside the nuclei or in nuclei with >2 spots were discarded. Spots in the promoter channel were matched to their nearest neighbor in the enhancer channel using Scipy linear sum assignment [78] and the Euclidean 3D distance between the 2 channels for each pair was calculated.

For STED images, the STED detail images were filtered for images containing 1 spot in the promoter channel, and the number of targeted enhancers in the enhancer channel. The Euclidean 3D distance between the promoter and all its enhancers was calculated. The expected inaccuracy of pairwise distance measurements is below 10nm [65].

To align sequential RNA and DNA FISH data, we detected fluorescent beads in the 488nm channel in the overview stacks of both rounds and converted their pixel coordinates to global coordinates by combining them with the stage position of the image. To match beads between rounds, we assigned each bead a descriptor based on the vectors to its nearest neighbors expressed in a local coordinate system via QR-factorization, similar to [79]. After matching beads based on descriptor distance, a similarity transform between the images was estimated using RANSAC. Finally, we applied this transform estimated from beads to the (global) coordinates of detected FISH spots to enable measurement of distances between RNA and DNA FISH signals. The mean alignment error per image was ∼460 nm.

Code for image analysis is available at github.com/CALM-LMU/enhancer-promoter.git or Zenodo: doi.org/10.5281/zenodo.14698025.

### Quantification of localization precision

To quantify localization precision in spinning disk measurements, we recorded 29 stacks of FISH samples of mESCs with a 20 kb region overlaping the *Nanog* promoter alternatingly stained with both ATTO565 and STAR635P (Supplementary Fig. S4A). Spots were detected in both channels using our standard pipeline of RS-FISH followed by Gaussian fitting. Affine chromatic aberration correction matrices were applied to the coordinates of the STAR635P channel. After correction, the x, y, and z components of vectors between matched spots (nearest neighbors were matched using linear assignment) are symmetrically distributed around zero with standard deviations of around 40 nm in z and 30 nm in x and y (Supplementary Fig. S4B). This inaccuracy estimation combines localization inaccuracies in both channels and remaining chromatic aberrations and due to the symmetric distribution around zero it should not introduce systematic bias, as it applies equally to all distance measurements. We quantified localization precision of STED measurements as part of a previous study [65] and found the expected inaccuracy of pairwise distance measurements to be below 10nm.

### Simulations of statistical power

To assess the effects of localization imprecision on statistical power, we performed simplified simulations in which we sampled two sets of vectors with random orientations and lengths drawn from a Maxwell–Boltzmann distribution (with scale parameter a picked so mean lengths match average distances observed in data). These vectors would correspond to the position of one of two FISH signals with the other one placed at the origin. We then simulated localization uncertainty by adding random “noise” vectors from a multivariate Normal distribution to each vector. The magnitudes of all resulting vectors were calculated, and the two distance distributions were checked for significant differences via a two-sided Wilcoxon rank-sum test. By repeating this procedure 1000 times and counting the fraction of significant tests, we get a measure of the statistical power (Supplementary Fig. S4C).

Using sample sizes and localization uncertainties approximately matching experimental data, we performed a grid search of various mean distances and differences in mean. To match spinning disk experiments, we use two equal samples of size 1500 and noise standard deviation of 40 nm. We estimate high statistical power at points in the parameter space corresponding to observed significant naive-primed differences. It should be noted that even when we sampled our noise vectors from a Normal distribution with nonzero mean (simulating uncorrected chromatic aberrations) or higher levels of inaccuracy, statistical power remained high, as both simulated distance distributions would be affected in the same way.

In conclusion, while remaining inaccuracies in the spot localization may skew the measured values, statistical significance of differences between two conditions/targets can still be determined with high power.

### Statistical analyses

Statistical analysis was performed using R (www.R-project.org/). Two-sided Wilcoxon rank sum tests were calculated for all comparisons. When comparing multiple groups, Benjamini–Hochberg (BH) false discovery rate correction was applied.

### Tri-C

For the Tri-C experiments, three independent biological replicates were performed as previously described [80, 81]. Briefly, 5 million cells per replicate were fixed in 5 ml culture medium using 2% formaldehyde (Thermo Fisher Scientific, 28908) at room temperature for 10 min while rotating. Formaldehyde was quenched with 125 mM ice-cold glycine, the cells were centrifuged at 4°C and 500 × *g* for 10 min and washed with cold PBS. The cell pellet was then resuspended in fresh lysis buffer (10 mM Tris, 10 mM NaCl, 0.2% Igepal CA-630, 1× cOmplete Protease Inhibitor cocktail), incubated at 4°C for 30 min and washed once with cold PBS. Restriction enzyme digestion was performed with 750 U DpnII (NEB, R0543S) at 37°C for 24 h. DpnII was subsequently inactivated at 65°C for 20 min and proximity ligation took place in 1× ligation buffer and 240 U T4 DNA ligase (Thermo Fisher Scientific, EL0013) at 16°C for 18 h. Ligation reactions were centrifuged at room temperature and 500 × *g* for 15 min, the pellets were resuspended in 300 μl of Tris–EDTA buffer (TE buffer; Sigma–Aldrich, 93283) and incubated with 3 U proteinase K at 65°C for 16 h, followed by an incubation with 7.5 mU of RNase (Roche, 11119915001) at 37°C for 30 min. DNA was subsequently extracted from the samples by mixing with equal volume phenol-chloroform-isoamyl alcohol mixture (Sigma–Aldrich, 77617) and centrifugation at room temperature and 12 600 × *g* for 10 min in prespun 5Prime light Phase Lock gel tubes (VWR, 733-2477). The upper aqueous layer was then mixed with 30 μl sodium acetate 3M, 1 μl GlycoBlue Coprecipitant (Thermo Fisher Scientific, AM9515) and ethanol 75% v/v at −20°C for 2 h. DNA was pelleted by centrifugation at 4°C and 21 000 × *g* for 30 min, washed with cold 70% ethanol, resuspended in TE buffer and concentration was determined with Qubit^TM^ 1× double-stranded DNA (dsDNA) Broad Range assay (Thermo Fisher Scientific, Q33266), according to the manufacturer’s instructions.

A total of 6.5 μg of DNA in 130 μl TE buffer were sonicated to fragments of 450 bp using a Covaris S220 Ultrasonicator (55 s; 10% duty factor; 140 W peak incident power; 200 cycles per burst) and fragments larger than 300 bp were size selected with 0.7× volume Mag-Bind TotalPure NGS beads (Omega Bio-tek, M1378-01). Four micrograms of each sample were indexed in duplicates (2 μg each) using the NEBNext Ultra II Library Prep kit for Illumina (NEB, E7645S). Briefly, samples were first incubated with 3 μl End Prep enzyme in 7 μl 10× End Prep buffer at 20°C for 45 min and 30 min at 65°C, followed by an incubation in 30 μl Ultra II Ligation Master Mix with 7 μl of NEBNext Adaptor and 1 μl Ligation Enhancer at 20°C for 30 min and a final incubation with 3 μl of the USER enzyme at 37°C for 30 min. Samples were then subjected to size selection with 1.8× volume Mag-Bind TotalPure NGS beads. Each sample was subsequently split in two indexing PCR reaction, performed with the Herculase II Fusion DNA polymerase kit (Agilent Technologies, 600677) by incubating 28.5 μl adaptor-ligated library, 5 μl NEB Universal primer, 5 μl NEB index primer, 10 μl Herculase II 5× buffer, 0.5 μl dNTPs and 1 μl Herculase II polymerase in a thermocycler at initial 98°C for 30 s, followed by 98°C for 10 s, 65°C for 30 s, 72°C for 30 s for 7 cycles and 72°C for 5 min. Samples were then subjected to clean-up with 1.8× volume Mag-Bind TotalPure NGS beads.

For the enrichment of fragments containing the viewpoints of interest, two subsequent hybridization reactions were performed using the KAPA HyperCapture Reagent kit (Roche, 9075828001) with biotinylated single-stranded DNA (ssDNA) probes complementary to the regions of interest. The ssDNA probes used for the Capture reactions were 100 nt long (Supplementary Table S7), designed using the oligo 0.2.0 python package (oligo.readthedocs.io/en/latest/) and ordered from Integrated DNA Technologies as two xGen^TM^ MRD Hybridization panels. Probes targeting positions found at a short distance on the genome were not included in the same panel. For the first hybridization reaction, 2 μg of each uniquely indexed library were combined in 1:1 mass ratio with 5 μl per library mouse Cot-1 DNA (Thermo Fisher Scientific, 18440016) and desiccated at 45°C in a vacuum centrifuge. The DNA pellet was then resuspended in 6.7 μl per library Universal Enhancing Oligonucleotides, 14 μl per library of Hybridization buffer, 6 μl per library Hybridization Component H and 4.5 μl per library diluted biotinylated oligonucleotides (2.9 nM per probe) and incubated at 95°C for 5 min and at 47°C for 72 h.

Pulldown of the viewpoint-containing fragments was performed with 51 μl of M-270 streptavidin Dynabeads (Thermo Fisher Scientific, 65306) per library and using the KAPA HyperCapture Reagent kit (Roche, 9075828001). Briefly, the beads were washed three times with 1× Bead Wash buffer at 47°C and incubated with the entire hybridization reaction at 47° and 600 rpm for 45 min, for the probes to bind to the beads. The bead-bound DNA was washed twice with 100 μl 1× Stringent Wash buffer per library at 47°C and 600 rpm for 5 min and consecutively with 100 μl 1× Wash buffer I, II, and III per library at room temperature for 1 min each time, resuspended in 40 μl per library PCR-grade water and subjected to clean-up with 90 μl per library Mag-Bind TotalPure NGS beads. Bead-bound DNA was PCR-amplified with 25 μl per library of 2× KAPA HiFi HotStart ReadyMix and 5 μl per library Post-Capture PCR Oligos as follows: initial 98°C for 45 s, followed by 98°C for 15 s, 60°C for 30 s, 72°C for 30 s for 14 cycles, and 72°C for 60 s. DNA was then subjected to cleanup with 90 μl per library Mag-Bind TotalPure NGS beads. All the captured DNA was used for a second capture reaction performed as described above but using the volumes for a single library and hybridization was performed for 24 h. Quality control was performed using D1000 TapeStation (Agilent Technologies, 5067-5583 and 5067-5582), Qubit^TM^ 1× dsDNA Broad Range assay (Thermo Fisher Scientific, Q33266) and fragment analyzer according to the manufacturers’ instructions. The libraries were sequenced with 300 cycles of paired-end reads on the Illumina NextSeq 2000 platform. Data analysis was performed using the CapCruncher pipeline [82] (github.com/sims-lab/CapCruncher) in capture mode against the mm10 genome assembly. Custom python scripts were used for the extraction of the reads with two or more *cis* reporter fragments, for the calculation of multiway interaction counts and the interaction matrix plotting [83].

## Results

### Changes in pairwise E–P distances during naive to primed transition 

To investigate whether E–P distances change when genes are upregulated/downregulated during differentiation, we utilized an *in vitro* model of the transition from naive to primed pluripotency in mESCs [84] (Fig. 1A and B). This transition is characterized by large gene expression changes and extensive rewiring of E–P contacts [43–46]. Cell state identities were validated via qRT-PCR and immunostaining of key marker genes (Supplementary Fig. S1A and B). We identified putative enhancers across both naive and primed states using the “Activity-by-contact model” [60]. From this set, we selected genes with at least three distinct enhancers, either functionally validated or predicted using the ABC model (Supplementary Table S3, Fig. 1C, and Supplementary Fig. S2). 

Target regions were visualized via oligonucleotide-based FISH (DNA oligoFISH) and imaged using two-color spinning disk confocal microscopy (Fig. 1D). For pairwise measurements of the *Nanog* –5 enhancer, the enhancer and promoter were visualized using nanoscopy-compatible oligonucleotides with dyes in variable arrays (NOVA FISH) [41] and imaged using automated STED super-resolution microscopy. Spots were detected with subpixel-localization accuracy using RS-FISH [76]. The subpixel localization in confocal images was refined using Gaussian fit (see the ‘Materials and methods’ section; Supplementary Fig. S4). To confirm that we have sufficient power to detect differences between naive and primed states, we performed simulations of statistical power (see the ‘Materials and methods’ section; Supplementary Fig. S4).

An ∼8.5× decrease in *Nanog* transcription (from 11% to 1.3% GAPDH transcription, Fig. 1B) was accompanied by nonsignificant differences (*P* >.05, two-sided Wilcoxon rank sum test with BH correction) in median 3D E–P distance (Fig. 1E). A similar trend was observed for E–P pairs of some measured genes (*Dppa3, Prdm14*). Notably, the largest, highly significant changes in E–P distance were observed between the *Sox2* promoter and the *Sox2* control region (SCR) (Δ_n→p _= 110 nm) as well as the *Sox2* promoter and a putative enhancer ∼1280 kb downstream (Δ_n→p _= 110 nm) (Supplementary Fig. S7A). Interestingly, for *Dnmt3a*, enhancer and promoter move significantly further apart, despite the gene being upregulated in the primed state (Supplementary Fig. S7A). We verified that these differences in E–P distance are not a result of a difference in nucleus size between the two cell types (Supplementary Fig. S5). Additionally, comparison of the selected targets with published live-cell measurements in mESCs [4, 85] confirmed that our DNA FISH procedure does not introduce major artifacts (Supplementary Fig. S6). Additionally, spatial distances for most E–P pairs seemed to be largely influenced by genomic distance (Supplementary Fig. S7B). These findings indicate that transcriptional changes during the naive to primed transition are gene specific and suggest that regulation does not universally require major alterations in 3D genome architecture.

### Changes in multiway E–P contacts during naive to primed transition

We then asked whether more complex 3D chromatin structures that may encompass some or all enhancers of one promoter (Fig. 2D) are enriched in naive and/or primed mESCs. Conventional chromosome conformation capture methods, such as Hi-C or Micro-Capture-C, are based on the detection of pairwise interactions and thus unable to detect such complex structures. We therefore performed Tri-C to capture *cis*-regulatory interactions at the *Nanog/Dppa3* locus (Fig. 2C and Supplementary Fig. S8). Tri-C interactions are depicted using viewpoint-specific contact matrices, which display the frequencies at which two chromatin fragments simultaneously interact with the viewpoint (Fig. 2B). Note that the regions across the diagonals from the viewpoints are in close genomic proximity to the viewpoint and are therefore expected to form strong interactions. Therefore, those regions have been grayed out from the contact matrixes [83].

At the *Nanog* locus in naive cells, we observe a weak enrichment of three-way contacts between the promoter and the −45 and −5 enhancers, as well as the −45, −5, and +60 enhancers (Fig. 2C). Interestingly, in primed cells where *Nanog* is downregulated, this enrichment of three-way contacts is lost.

To further investigate multiway interactions at a single-cell level, we focused on the example of *Nanog*, where we observed changes in multiway interactions via Tri-C. We imaged the promoter (labeled with one barcode) and its cognate enhancers (labeled with a second barcode) using STED super-resolution microscopy (Fig. 2E). The plots display a percentage of alleles, in which a certain number of enhancers are in proximity to the promoter simultaneously. Since it is unknown over which distances promoters and enhancers can exchange information, this information is shown for a range of different distance thresholds (Fig. 2F). For a visualization with an adjustable distance threshold see our data viewer: py.cafe/app/hoerldavid/promoter-enhancer-interactive-fig2. To avoid artifacts that might be caused by the different sensitivity of the hybridization procedure for the different enhancers, only images with all enhancers labeled were analyzed for Fig. 2F.

We observe that multiway E–P contacts, compared to pairwise contacts, are rare. For example, at the *Nanog* locus (−45 E, −5 E, promoter, +60 E; Fig. 2E) in naive cells, for an arbitrary distance threshold of 200 nm [86], pairwise E–P contacts occur in 55% of all alleles, while three-way contacts (P–E–E) occur in 25%, and four-way contacts (P–E–E–E) in only 3% of alleles (Fig. 2F and G). Interestingly, in the primed state where *Nanog* is downregulated, pairwise as well as multiway contact frequencies decrease by a few % for E–P distances below 300 nm. To a smaller extent, a reduction in three-way, but not four-way E–P contacts upon differentiation can also be observed for *Dppa3* (Supplementary fig. S9B and C). Together with Tri-C, these data show that downregulation of *Nanog* expression during the naive to primed transition is accompanied by a small reduction in multiway contact frequency.

### Effect of transcription on E–P distance

Since many genes are transcribed in bursts, we reasoned that transient changes in E–P distances associated with transcription could be hidden at a population level (Fig. 3A). To address this, we designed probes targeting introns of *Nanog* (Fig. 3B) and performed a sequential protocol starting with RNA SABER FISH against the intron, followed by DNA FISH against the –5, –45, and + 60 enhancers (Fig. 3C; see the ‘Materials and methods’ section). We assumed that the distance between the nascent RNA and the promoter can serve as a proxy for transcriptional timing, with shorter distances indicating a more recent transcription event.

For *Nanog*, alleles which were transcribed more recently show significantly decreased E–P distances (Fig. 3E). For example, when a nascent RNA is located within 0.25 µm of the promoter, the median E–P distance is about 50 nm shorter than when the RNA is within 0.5 µm, and about 90 nm shorter than when it is within 1 µm (*P* <.05, two-sided Wilcoxon rank sum test). A similar effect was also observed for *Dppa3* (Supplementary Fig. S10C). When the three labeled *Nanog* enhancers were ordered by proximity to the promoter, a similar trend was observed, although not significant, likely due to the limited sample size (Supplementary fig. S10D). Together, these data indicate that for *Nanog* and *Dppa3*, E–P distances change based on the transcriptional state of the alleles.

## Discussion

Enhancers play a key role in gene regulation during development, but the spatiotemporal interaction with their promoters is still under investigation. Here, we use oligoFISH, NOVA FISH, and SABER FISH, combined with various super-resolution microscopy techniques and Tri-C to investigate selected differentially expressed genes of mESCs in different states: during the transition from naive to primed pluripotency and after onset of transcription.

Our data suggest that changes in pairwise E–P distances during the naive to primed transition are gene- and enhancer-specific. For example, although *Nanog* E–P distances do not significantly change between states, the SCR moves significantly further away from its promoter (Δ ≈ 110 nm) when downregulated. Our findings at the investigated *Nanog* loci are consistent with Ohishi *et al.* (2025).

Furthermore, our Tri-C data suggest the presence of weak multiway E–P hubs at the *Nanog* locus, which disappear when *Nanog* is downregulated in the primed state. Formation of stronger hubs has previously been reported at the globin loci [87, 88] and for primed specific genes [43]. Such differences may reflect cell type and locus-specific effects. In line with the Tri-C data, our microscopy data show only minor differences between the naive and the primed state. A central limitation in the interpretation of microscopic E–P contact data is that measured contact frequencies do not account for spontaneously occurring interactions, necessitating appropriate normalization [85, 89]. By contrast, Tri-C and related proximity ligation assays provide data on the enrichment of contacts over background in a population of cells.

A key unresolved question in assessing E–P contacts is what counts as a functional contact. Different distance thresholds have been reported, with some models suggesting that sequential “contacts” may be required to trigger transcription [90, 91]. Moreover, the duration of a “contact” necessary to trigger an event is unknown. Therefore, further assumptions or even complex models are necessary to interpret absolute contact frequencies [85, 89]. At an arbitrary threshold of 200 nm, absolute pairwise contacts (P–E) at the *Nanog* locus occur in 55% of alleles, three-way contacts (P–E–E) in 25%, while four-way contacts (P–E–E–E) occur in only ∼3% of alleles. Interestingly, our data differ markedly from results of a recent preprint [86], reporting much lower multiway contact frequencies at the *Nanog* locus. The most likely explanations for this discrepancy are methodological and/or underlying biological differences.

Interpretation of FISH measurements requires careful consideration, since these measurements are prone to artifacts due to fixation and harsh sample preparation. To assess the robustness of our FISH procedure and subsequent data acquisition and—analysis steps, we compared our pairwise FISH data at the Fbn2 TAD and Sox2—SCR region with state of the art live-cell measurements in mESCs [4, 85] and found good concordance (Supplementary Fig. S6).

Our RNA-DNA FISH data show that E–P distances change with the transcriptional state, suggesting that such contacts are likely transient. *Nanog* and *Dppa3* regions in which transcription occurred recently, as determined by a nascent RNA closer to the promoter, display significantly shorter E–P distances (Fig. 3E). This is in agreement with a recent study by Ohishi et al 2024, which found that *Nanog* enhancers spend longer times in the proximity of actively transcribed promoters. However, a limitation of fixed-cell studies is that each cell captures only a single moment, “a snapshot”, within a continuous process. Since our measurements detect the mRNA but not the regulatory initiation event, it is unclear to what extent the observed 3D conformation reflects the state at transcriptional onset. Live-cell measurements at TAD boundaries have demonstrated the dynamic and transient nature of loop structures, a property that may also extend to E–P looping interactions [85]. For this reason, small or even absent changes in E–P distances of genes which are differentially expressed should not be over interpreted as in a population of cells only a fraction of alleles is transcribed. Here we show for the *Nanog* and *Dppa3* locus a short-lived E–P distance reduction correlated with transcription which is not detected in measurements of the whole population. This seems to be gene- and cell type-specific, as live-cell imaging studies have reported both correlations and lack of correlations between E–P distance and transcription, depending on the system examined [92].

In summary, our data show that during the naive to primed transition, most of the studied genes show no major changes in average E–P distances, even though expression levels change by several orders of magnitude. This suggests that regulation of many genes may not always depend on change in the underlying 3D genome architecture. At the *Nanog* locus, Tri-C data show a weak enrichment of multiway contacts in the naive state where the gene is highly expressed. This supports models proposing that multiway contacts play a role in transcriptional regulation. Since the required duration of pairwise or multiway contacts and the necessary spatial proximity remain elusive, our microscopy data can help parameterize quantitative models. Finally, we observe a correlation between the presence of a nascent transcript and a shortening of the corresponding E–P distances. This is in line with models where short E–P distances are stabilized transiently during transcription, possibly within a multiprotein complex or transcriptional condensate.

## Supplementary Material

gkaf1255_Supplemental_Files

## Data Availability

The FISH spot coordinates generated in this study have been deposited at doi.org/10.5281/zenodo.17115347. Imaging data is available upon reasonable request. The Tri-C data generated in this study have been deposited at GEO: GSE308397. The reanalyzed data used for enhancer calling can be found at GEO: GSE131556 (ATAC-seq), GEO: GSE156261 (H3K27ac ChIP-seq), GEO: GSE131556 (RNA-seq) [48] and GEO: GSE124342 (Hi-C) [61]. The code used for analyzing and processing the images can be found at: doi.org/10.5281/zenodo.17123359. The STED automation code can be found at: doi.org/10.5281/zenodo.14627119.
