# Supplementary material for: Nanoscale dynamics of enhancer–promoter interactions during exit from pluripotency
Source: Nucleic Acids Res. 2025 Dec 8;53(22):gkaf1255. doi: 10.1093/nar/gkaf1255 (PMC12684394; doi:10.1093/nar/gkaf1255)
Supplement: gkaf1255_Supplemental_Files [file gkaf1255_supplemental_files.zip › supplementary_figures.pdf]

SUPPLEMENTARY FIGURES for

Nanoscale Dynamics of Enhancer-Promoter Interactions during Exit from Pluripotency

Authors:
Gabriela Stumberger, David Hörl, Dimitra Tsouraki, Clemens Steinek,
A. Marieke Oudelaar, Heinrich Leonhardt, Hartmann Harz

Correspondence:
harz@biologie.uni-muenchen.de, h.leonhardt@biologie.uni-muenchen.de

This PDF includes:

Supplementary Figure S1 (related to Methods)
Supplementary Figure S2 (related to Figure 1)
Supplementary Figure S3 (related to Methods)
Supplementary Figure S4 (related to Methods)
Supplementary Figure S5 (related to Figure 1)
Supplementary Figure S6 (related to Figure 1)
Supplementary Figure S7 (related to Figure 1)
Supplementary Figure S8 (related to Figure 2 A-C)
Supplementary Figure S9 (related to Figure 2 D-F)
Supplementary Figure S10 (related to Figure 3)

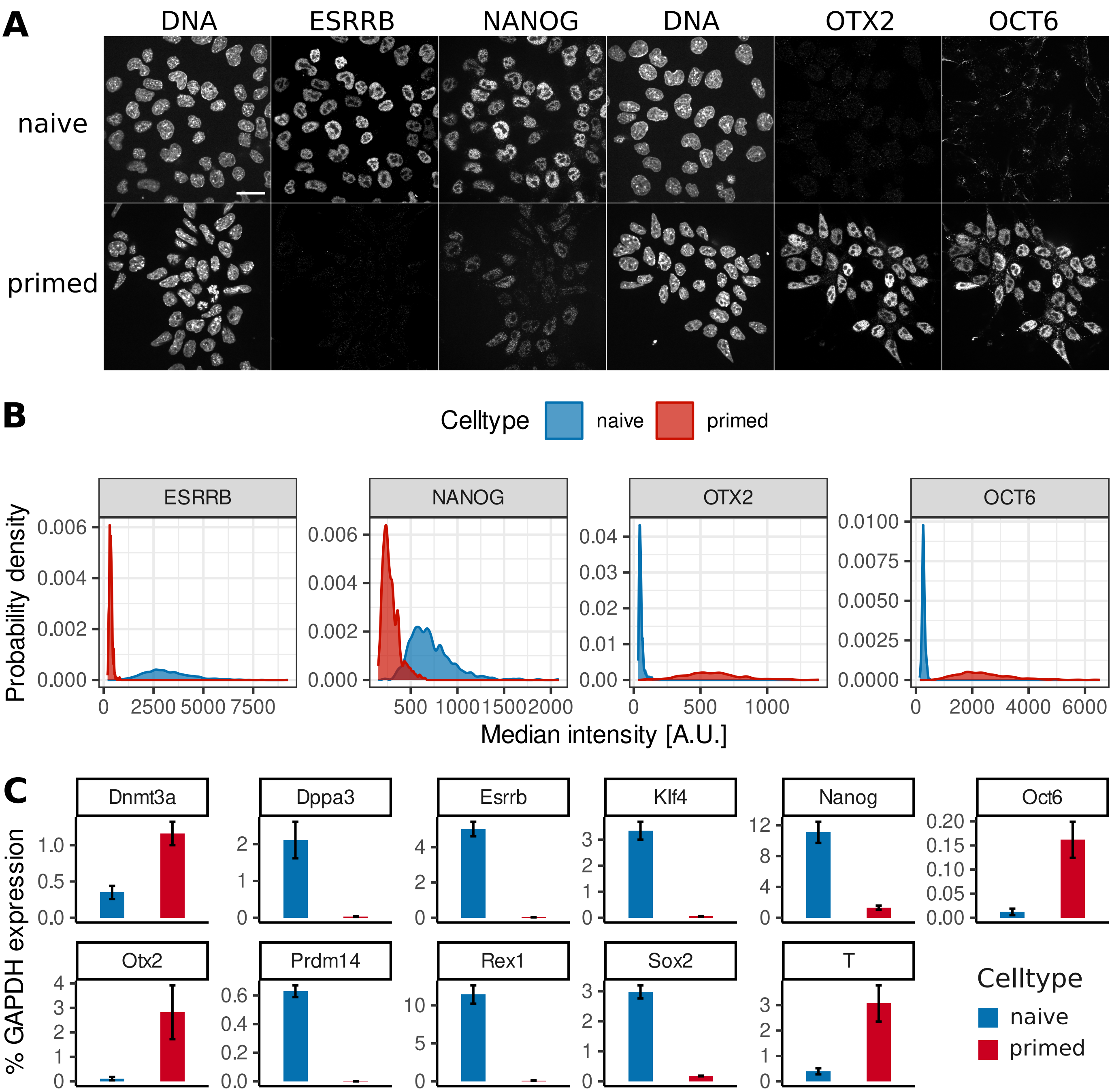

Supplementary Figure S1: Verification of naive -> primed system.
(A) Immunofluorescence of naive (ESRRB, NANOG) and primed (OTX2, OCT6) differentiation markers. Scale bar represents 20 µm. (B) Distribution of marker gene expression in naive (blue) and primed (red) cell nuclei. There is a clear decrease in ESRRB and NANOG expression and an increase in OTX2 and OCT6 expression as cells transition from naive to primed. ESRRB, NANOG: n_{naive}=665, n_{primed}=455; OTX2, OCT6: n_{naive}=680, n_{primed}=591. (C) mRNA expression levels of target makrer genes in naive (blue) and primed (red) mouse embryonic stem cells were measured via quantitative real-time PCR at 0 h (naive) and after 7 days (primed) of differentiation. Expression is represented as % GAPDH expression (median ± SEM, n=5 biological replicates).

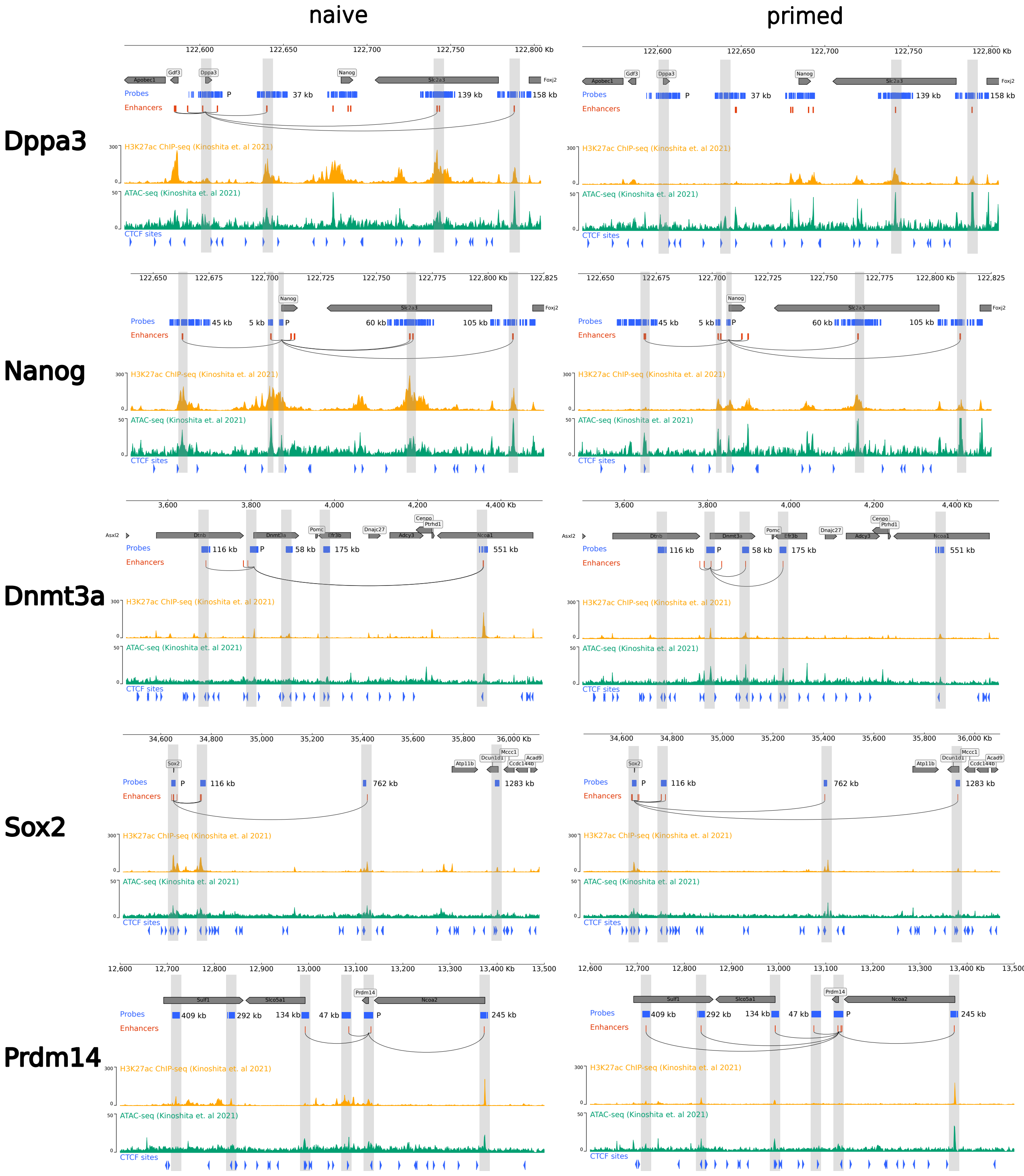

Supplementary Figure S2: Genomic data for target regions in naive and primed cells. For each gene and cell state, from top to bottom: DNA oligoFISH probes against promoter and selected enhancers (blue), all predicted enhancers (red), connection between promoter and its predicted enhancers (black arcs), H3K27ac ChiP signal (yellow, from Kinoshita et al. 2021), ATAC-seq signal (green, from Kinoshita et al. 2021), CTCF binding motifs (blue arrows) and targeted enhancer regions (vertical gray stripes).

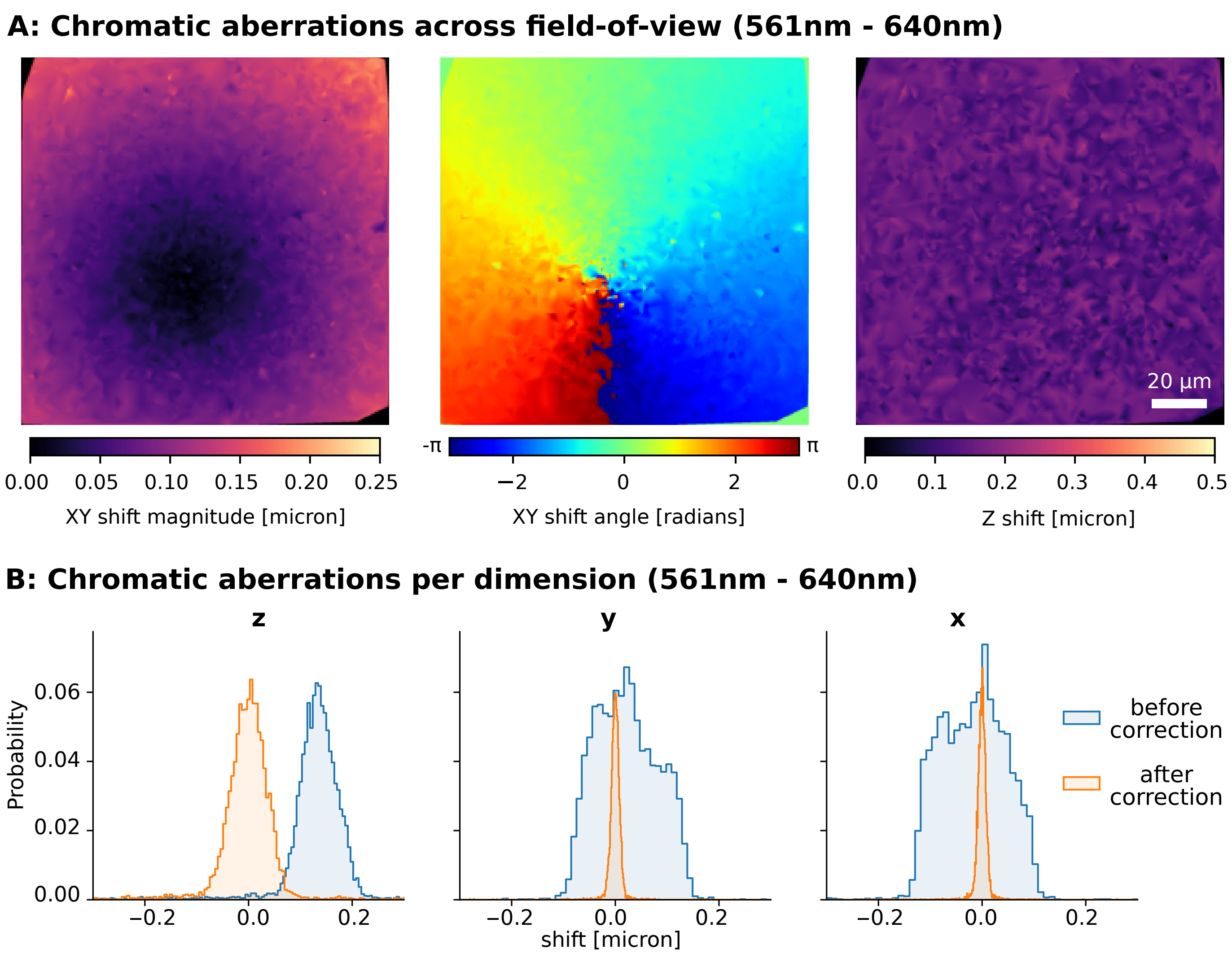

Supplementary Figure S3: Characterization of chromatic aberrations between the 561nm and 640nm channels of our spinning disk microscope. (A) Shifts across the field-of-view interpolated from matched Tetraspeck beads. (B) Histograms of shifts (difference of coordinates of matched beads) per dimension of the same dataset. Corrected coordinates were calculated using a leave-one-out cross validation scheme on the 11-image calibration dataset.

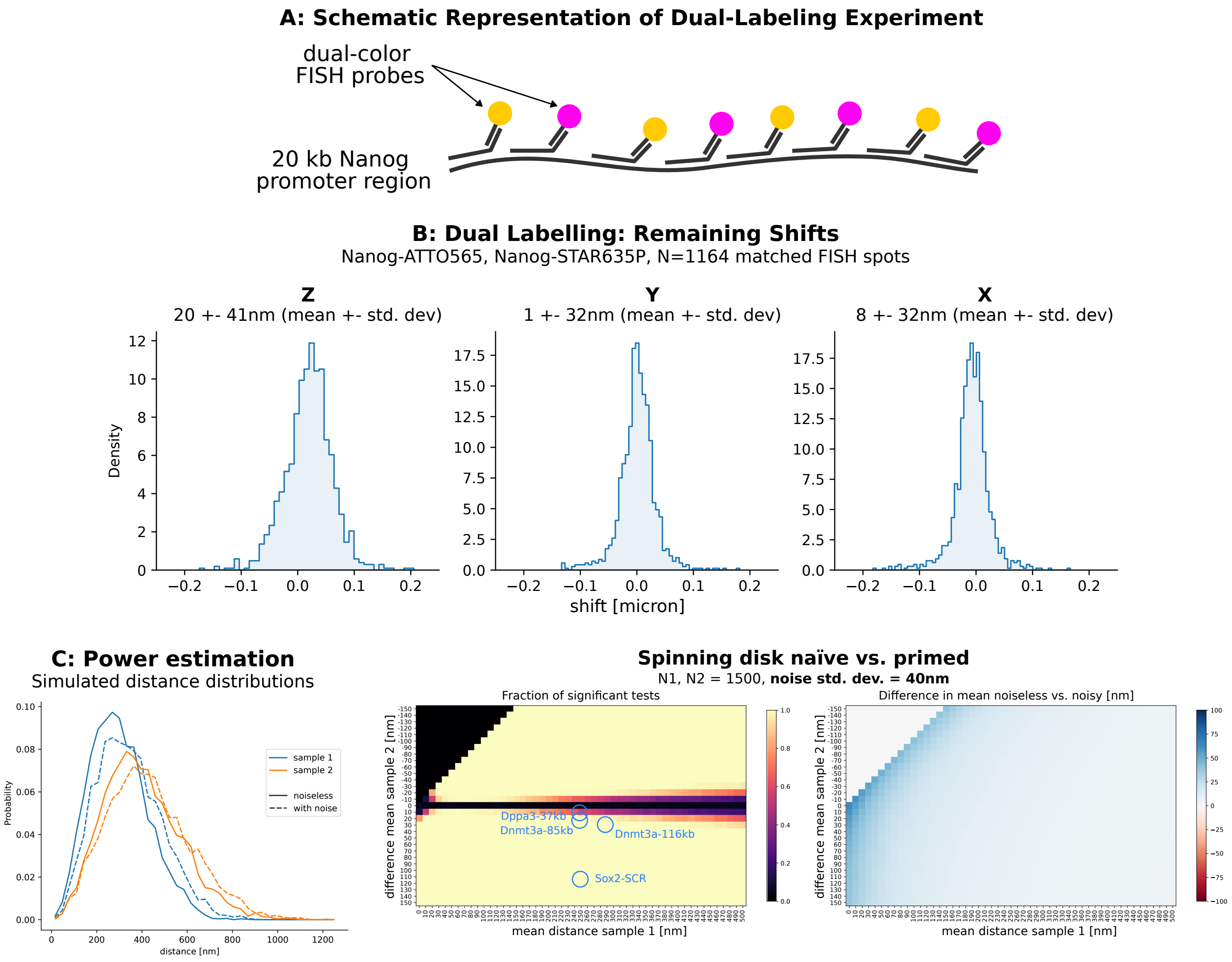

Supplementary Figure S4: (A) Schematic representation of dual-labeling experiment: a 20 kb region overlapping the Nanog promoter was labelled with alternating ATTO565 and STAR635P FISH probes. (B) Distance distributions of matched FISH spots in two channels in a dual-labelling experiment per dimension. (C) Statistical power estimation via repeated simulations of pairs of distance distributions. Middle: Fraction of 1000 repetitions that resulted in significant differences for various combinations of mean distances in sample 1 and difference in mean in sample 2. Combinations that would result in negative distances are skipped. Combinations of average distances that show significant differences in observed data are highlighted in blue. Right: Mean differences in distance between simulated vectors with and without noise.

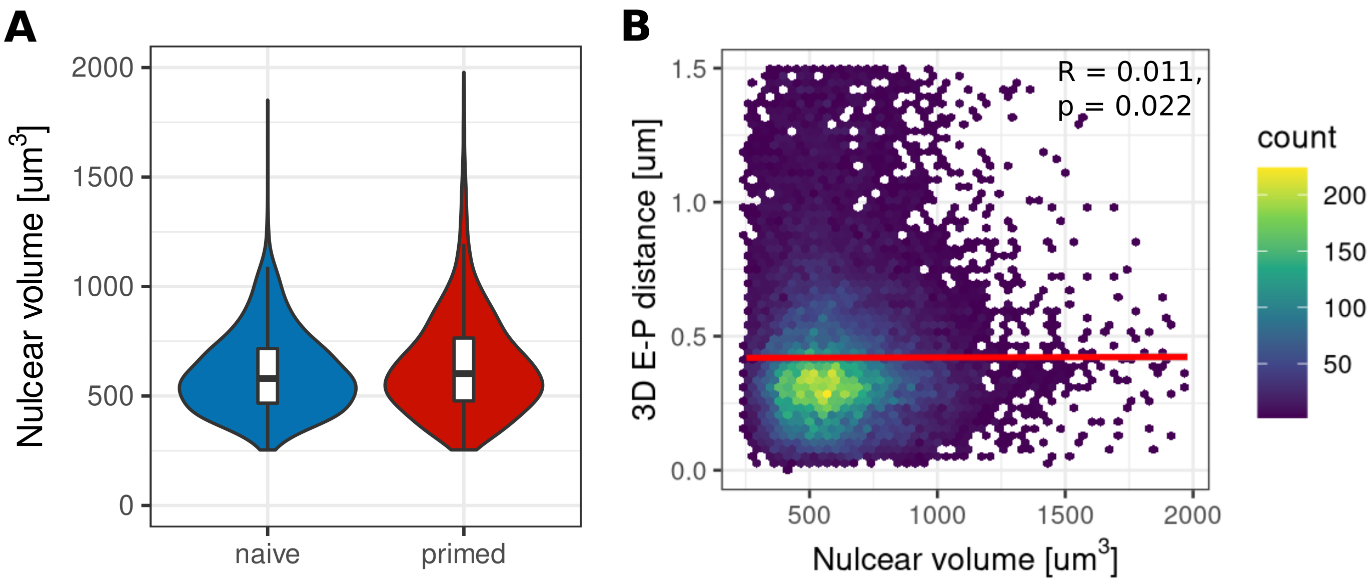

Suppplementary Figure S5: Relationship between nuclear volume and enhancer-promoter (E-P) distance. (A) Nuclear volume [µm³] distributions in naive (blue) and primed (red) cells (n_{naive}=27167, n_{primed}=23970, from all nuclei in Fig 1 F, measured with spinning disk confocal micorscope). (B) There is no notable correlation between nuclear volume and E-P distances (Spearman's Rank Correlation, R=0.011, p=0.022; n= 51137, over 2 celltypes and 3 biological replicates).

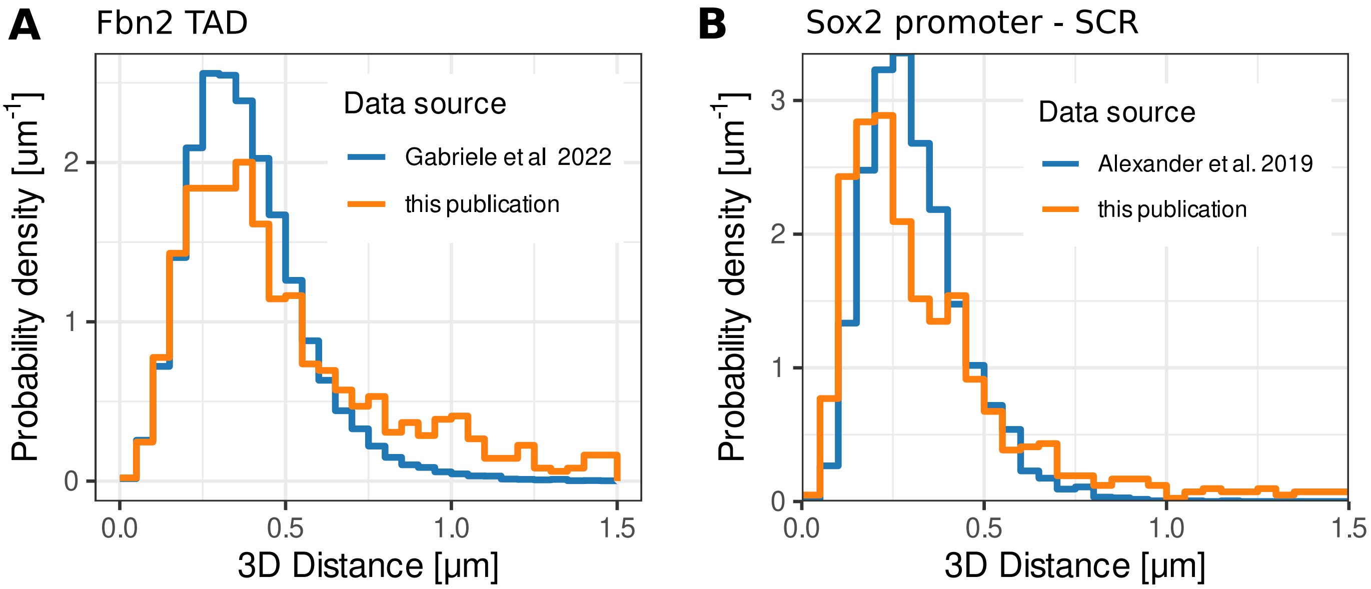

Supplementary Figure S6: Comparison of FISH and live cell measurments at sleected loci. (A) 3D distances at the Fbn2 locus for our DNA FISH (orange) and live cell measurements from Gabriele et al. 2022 (blue). $n_{\text{this publication}}$=979, $n_{\text{Gabriele et al. 2022}}$=46163. (B) 3D distances between the Sox2 promoter and the SCR enhancer region for our DNA FISH (orange) and live cell measurements from Alexander et al. 2019 (blue). $n_{\text{this publication}}$=831, $n_{\text{Alexander et al. 2019}}$=4380.

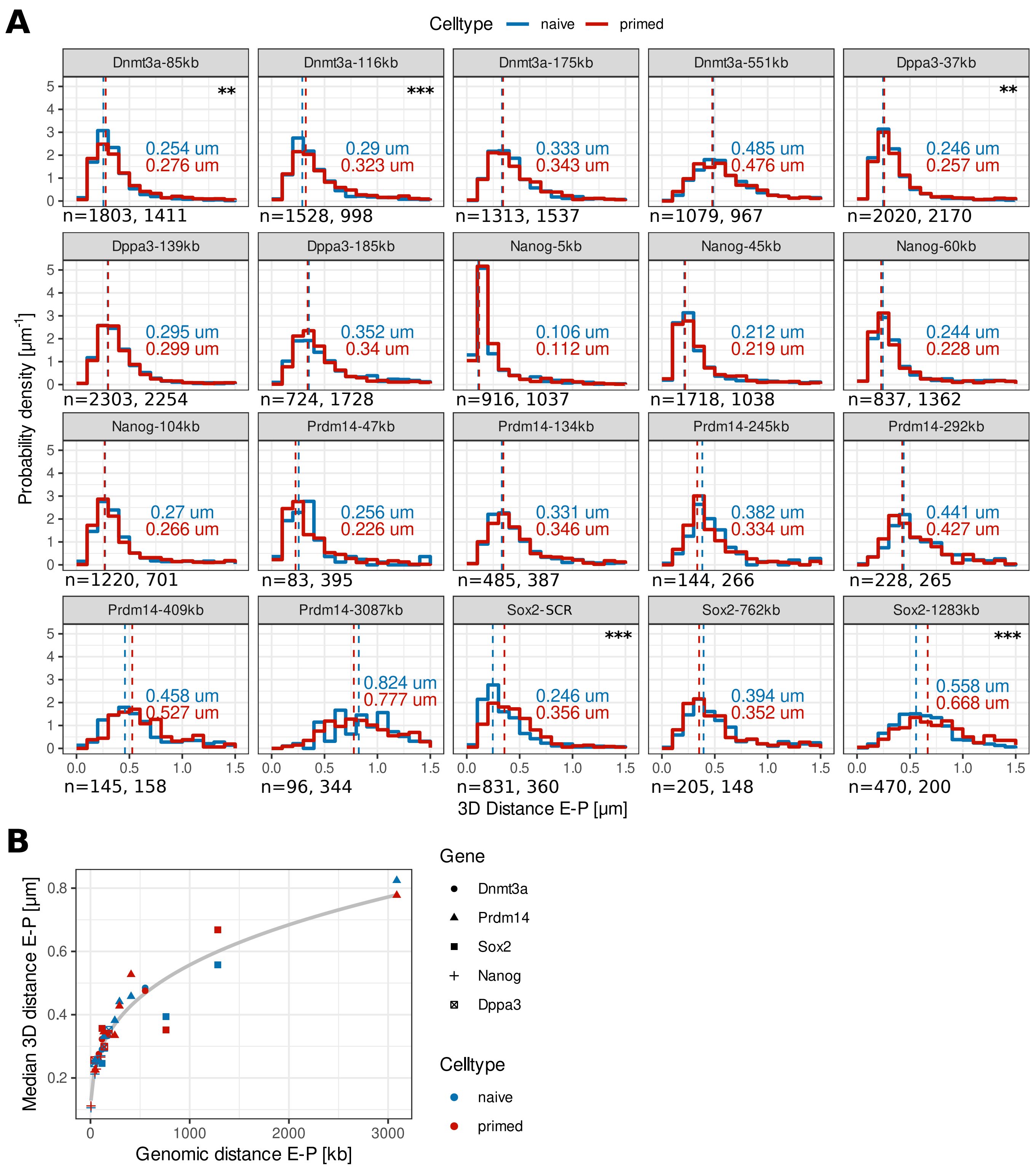

Supplementary Figure S7: Summary of enhancer-promoter (E-P) distances in developmental enhancers in naive and primed cells. (A) 3D distance [μm] distributions of promoters and their corresponding enhancers in naive (blue) and primed (red) cells. Dashed line and number next to histogram represent the median distance. Significant differences between naive and primed indicated by: * $p \leq 0.05$, ** $p \leq 0.01$, *** $p \leq 0.001$ (Wilcoxon rank sum test, Benjamini-Hochberg FDR correction). (B) Median 3D E-P distance [μm] as function of genomic distance [kb] for 5 genes. 3D distance for most E-P pairs is largely influenced by genomic distance. The curve was fitted to the data using a power law model: $y = a*x^b+c$, with estimated coeficients: a=0.63, b=0.31,c=0.024.

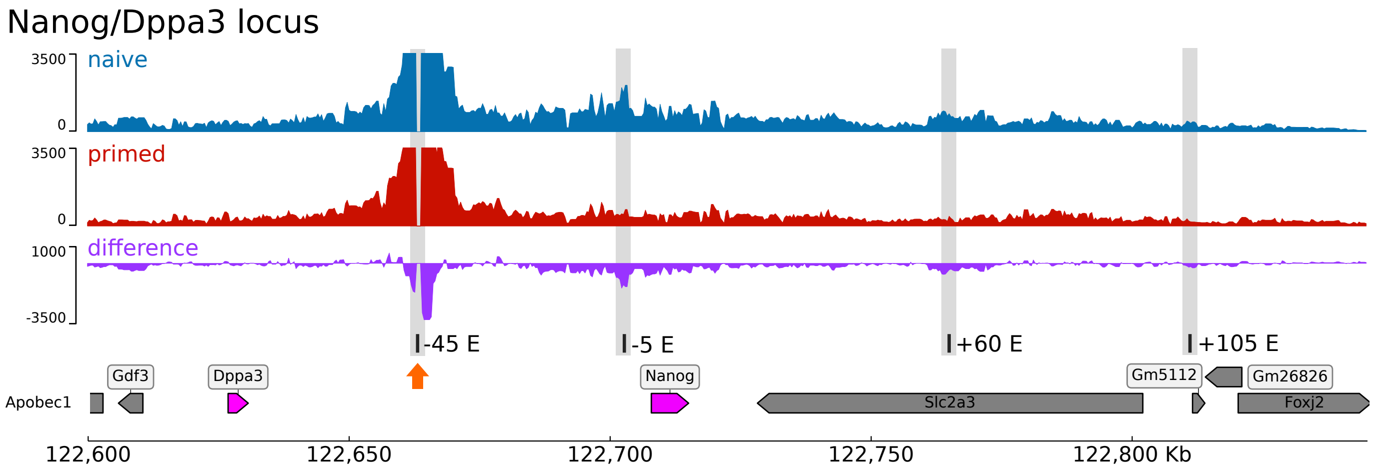

Supplementary Figure S8: Tri-C Pairwise interactions at the Nanog/Dppa3 locus. Naive cells are shown in blue, primed cells in red and the difference between the two (naive - primed) in purple. The viewpoint (Nanog -45 E) is indicated with an orange arrow.

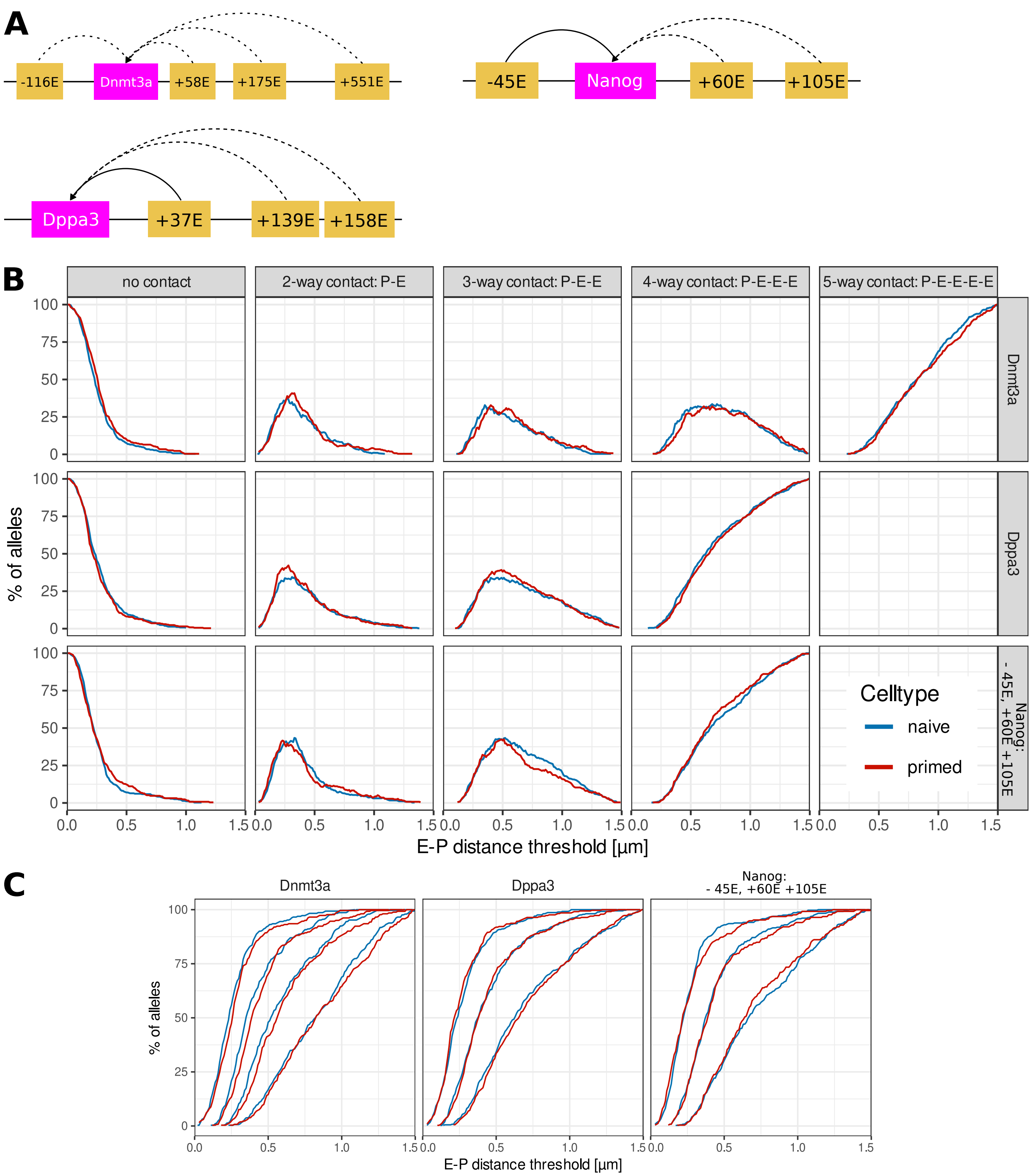

Supplementary Figure S9: Multiway E-P contacts during the naive to primed differentiation. (A) Schematic representation of measured interactions at Dnmt3a, Dppa3 and Nanog loci. (B) Interaction frequencies between a promoter (P) and different numbers of enhancers for a range of contact thresholds [μm]. Naive cells are plotted in blue, primed in red. Dnmt3a: n_n=292, n_p=262; Dppa3: n_n = 371, n_p =334; Nanog (-45,+60E,+105E): n_n=357, n_p=252 over 3 replicates. (C) % of detected alleles with at least 1, 2, 3 or 4 enhancers for a range of contact thresholds [μm]. For n see (B).

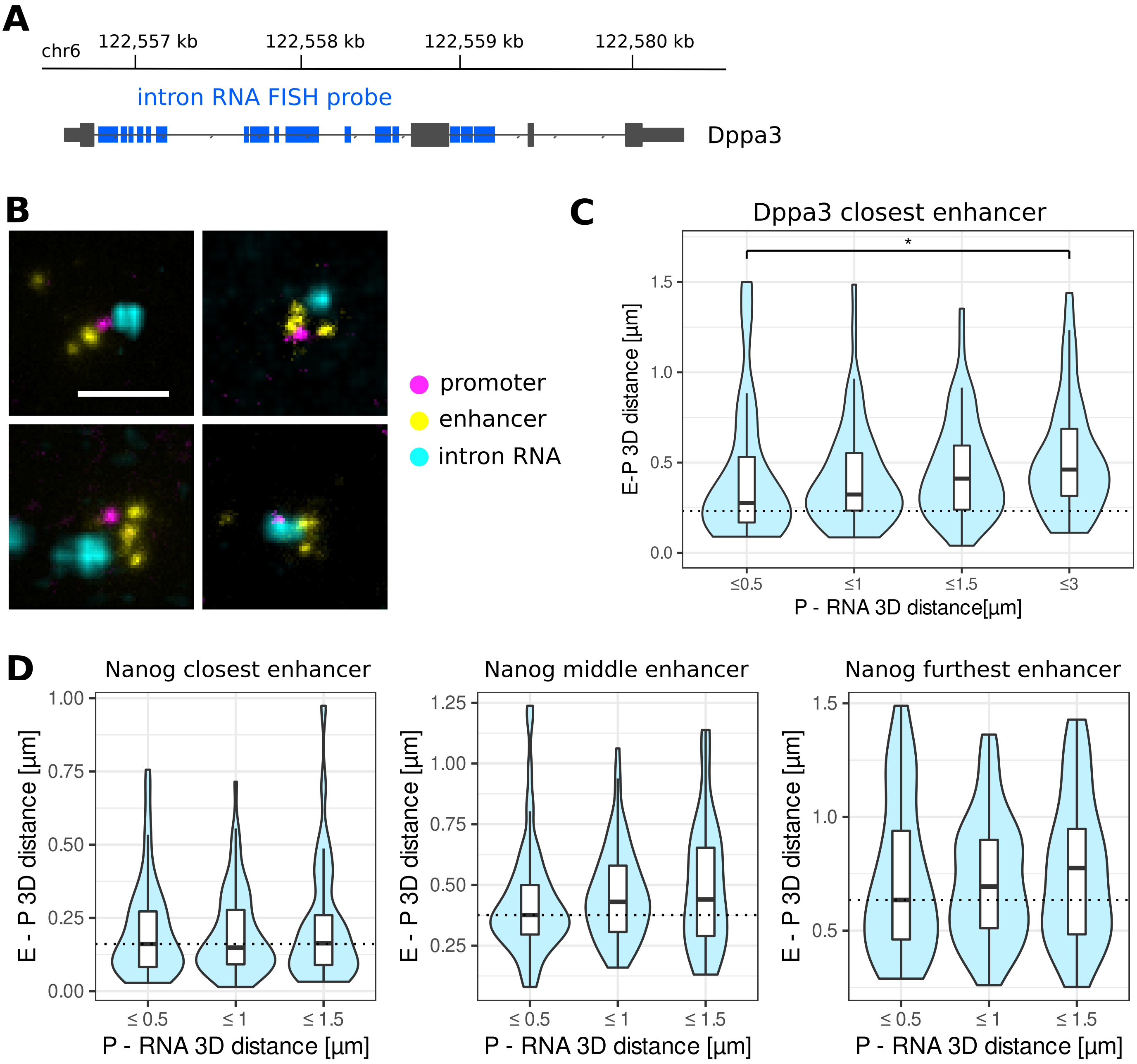

Supplementary Figure S10: (A) Shorter enhancer - promoter (E - P) distances correlate with active transcription. (A) Genomic location of Nanog intron RNA FISH probes. (B) STED microscopy images (maximum intensity projections) of promoter (magenta), enhancers (yellow) and intron RNA (cyan) for Dppa3. Scale bar represents 1 µm. (C) Dppa3 E-P 3D distance, based on distance of promoter to closest nascent RNA. ($p < 0.05$: *, two-sided Wilcoxon rank sum test). $n_{\leq 0.5} = 21$, $n_{\leq 1} = 59$, $n_{\leq 1.5} = 60$, $n_{\leq 3} = 109$ over 3 biological replicates. (D) E - P 3D distances of Nanog's closest middle and furthest enhancer, based on the distance of the promoter to the closest nascent RNA. $n_{\leq 0.5} = 57$, $n_{\leq 1} = 72$, $n_{\leq 1.5} = 35$ over 3 biological replicates.
